# African American Rural Caregiving: Assessing Needs and Identifying Solutions

**DOI:** 10.1093/geroni/igaf122.2972

**Published:** 2025-12-31

**Authors:** Laura Ainsworth, Scott Wilks, Rhonda Bardales

**Affiliations:** Louisiana State University, Baton Rouge, Louisiana, United States; Louisiana State University, Baton Rouge, Louisiana, United States; Louisiana State University, Baton Rouge, Louisiana, United States

## Abstract

Uncompensated caregiving for Americans with Alzheimer’s disease and related dementias (ADRD) is valued at $339.5 billion annually. With the national rise in older adults reaching 73 million by 2030, the prevalence of individuals with ADRD and their care partners also will rise. Underrepresented care partners (e.g., ethnic minority and non-urban residents) face caregiving burdens unique to cultural identities and geographical contexts. Yet, a dearth of literature exploring their experiences is glaring. The purpose of this study is to redress this gap, to gain a qualitative, meaningful understanding of caregiving perceptions and experiences among rural African American (RAA), ADRD care partners. Three separate, 2-hour focus groups were conducted with RAA ADRD care partners (N = 31) in south Louisiana to identify thematically specific caregiving needs and perceptions of resources to alleviate said needs. Prior to group discussions, participants completed a questionnaire with demographics and measures of anxiety, depression, and social isolation. Constructivist grounded theory methods were used to analyze narrative group data to construct concepts based on care partner perspectives, noting consistent themes. One-quarter of the sample reported symptoms of anxiety and depression;15% reported social isolation. The following themes were identified: lack of household role balance; negativity toward caregiving duties; lack of self-care; hardships exclusive to ethnic and rural cultural differences; and technology needed to lessen strain. This study revealed the daily struggles faced by an underresearched group of ADRD care partners. Highlighting technological innovations to address their struggles faced revealed a potential direction for African American, ADRD care partners in rural communities.

